# Efficacy of Phage- and Bacteriocin-Based Therapies in Combatting Nosocomial MRSA Infections

**DOI:** 10.3389/fmolb.2021.654038

**Published:** 2021-04-29

**Authors:** Lauren Walsh, Crystal N. Johnson, Colin Hill, R. Paul Ross

**Affiliations:** ^1^School of Microbiology, University College Cork, Cork, Ireland; ^2^APC Microbiome Ireland, University College Cork, Cork, Ireland; ^3^Teagasc Food Research Centre, Moorepark, Cork, Ireland

**Keywords:** bacteriophage, endolysins, bacteriocins, MRSA, nosocomial environment

## Abstract

*Staphylococcus aureus* is a pathogen commonly found in nosocomial environments where infections can easily spread - especially given the reduced immune response of patients and large overlap between personnel in charge of their care. Although antibiotics are available to treat nosocomial infections, the increased occurrence of antibiotic resistance has rendered many treatments ineffective. Such is the case for methicillin resistant *S. aureus* (MRSA), which has continued to be a threat to public health since its emergence. For this reason, alternative treatment technologies utilizing antimicrobials such as bacteriocins, bacteriophages (phages) and phage endolysins are being developed. These antimicrobials provide an advantage over antibiotics in that many have narrow inhibition spectra, enabling treatments to be selected based on the target (pathogenic) bacterium while allowing for survival of commensal bacteria and thus avoiding collateral damage to the microbiome. Bacterial resistance to these treatments occurs less frequently than with antibiotics, particularly in circumstances where combinatory antimicrobial therapies are used. Phage therapy has been well established in Eastern Europe as an effective treatment against bacterial infections. While there are no Randomized Clinical Trials (RCTs) to our knowledge examining phage treatment of *S. aureus* infections that have completed all trial phases, numerous clinical trials are underway, and several commercial phage preparations are currently available to treat *S. aureus* infections. Bacteriocins have primarily been used in the food industry for bio-preservation applications. However, the idea of repurposing bacteriocins for human health is an attractive one considering their efficacy against many bacterial pathogens. There are concerns about the ability of bacteriocins to survive the gastrointestinal tract given their proteinaceous nature, however, this obstacle may be overcome by altering the administration route of the therapy through encapsulation, or by bioengineering protease-resistant variants. Obstacles such as enzymatic digestion are less of an issue for topical/local administration, for example, application to the surface of the skin. Bacteriocins have also shown impressive synergistic effects when used in conjunction with other antimicrobials, including antibiotics, which may allow antibiotic-based therapies to be used more sparingly with less resistance development. This review provides an updated account of known bacteriocins, phages and phage endolysins which have demonstrated an impressive ability to kill *S. aureus* strains. In particular, examples of antimicrobials with the ability to target MRSA strains and their subsequent use in a clinical setting are outlined.

## Introduction

The current COVID-19 pandemic has revealed just how vulnerable humankind is to infectious agents where natural selection and mutations give rise to new pathogens for which we have few to no therapeutic solutions. Antimicrobial resistance (AMR) is another global public health crisis exacerbated by the lack of discovery of novel antibiotic agents and absence of investment and innovation in pipelines by the pharmaceutical industr*y. Staphylococcus aureus* infections can cause a range of ailments from skin conditions to potentially fatal diseases such as invasive endocarditis and sepsis (Lowy, 1998; Chambers, 2001). The emergence of antibiotic resistant bacteria began with penicillin resistant *S. aureus* (Enright et al., 2002). This threat was more or less overcome by the introduction of additional, antibiotics, such as methicillin, as alternative therapeutics to combat penicillin resistant *S. aureus*. However, following 2 years of successful treatment, methicillin resistant *S. aureus* (MRSA) emerged and has since developed into a global crisis (Enright et al., 2002). Indeed, antimicrobial resistance is a significant threat to the health of the human population, with predictions that by 2050 antimicrobial resistance will claim 10 million deaths per year (Hu et al., 2020). Antibiotic resistance not only has dire consequences for health but also increases costs associated with healthcare (European Centre for Disease Prevention and Control [ECDC], 2014) and has downstream consequences for the environment and economy.

Methicillin resistant *S. aureus* specifically has become a pathogen commonly found in nosocomial environments (Tiemersma et al., 2004; Weiner-Lastinger et al., 2020). Although medical devices and commonly used surfaces may mediate the transfer of MRSA, the primary vectors of transmission of MRSA are the patients themselves and the personnel they contact. In clinical environments, ointment containing the antibiotic mupirocin is commonly used to eradicate staphylococcal nasal carriage. While this type of therapeutic has been shown as an effective form of prevention and treatment, resistance does develop (Fenton et al., 2010a). Vancomycin is also a commonly used antibiotic to treat MRSA infection, however, vancomycin resistant *S. aureus* (VRSA) has now also become a prominent issue in the nosocomial environment (Horne et al., 2009). New antibiotics are being developed to fight against MRSA infection, such as ceftaroline which has been commercially available since 2012. Numerous other antibiotics have undergone phase III clinical trials including dalbavancin (NCT01339091), oritavancin (NCT01252719) and nemonoxacin (NCT02205112) (Table 1) (Pasberg-Gauhl, 2014). Although new antibiotics are being developed their susceptibility to bacterial resistance still remains a substantial problem for human health. Burn wound victims are particularly susceptible to MRSA infection in the nosocomial environment (Goudarzi et al., 2019). This comes as a result of a reduced immune response from the patient and disruptions to the physical barriers protecting the skin from colonization, resulting in a poor prognosis for burn wound patients infected with MRSA (Goudarzi et al., 2019). For these reasons, outbreaks of MRSA infections in burn units often have catastrophic results.

**TABLE 1:** Current antibiotic treatment of MRSA and newly developed antibiotics to treat MRSA.

Antibiotic	MIC for *S. aureus*	References
Vancomycin	0.25 - 1 mg/L	LaPlante, 2007
Mupirocin	0.94 - 1024 mg/L	LaPlante, 2007
Ceftaroline	1 mg/L	Jones et al., 2010
Dalbavancin	0.06 mg/L	Smith et al., 2015
Oritavancin	0.06 mg/L	Mendes et al., 2014
Nemonoxacin	<0.3 – 2 mg/L	Chen et al., 2009

While *S. aureus* infections have clear consequences on public health, deleterious effects are also observed in the dairy industry. *S. aureus* infections, including those of MRSA, are one of the main causative agents of mastitis, both in human and bovine populations (Field et al., 2016). Not only is this a threat to the health of humans and lactating animals but it also affects the quality of milk produced while increasing the burden of costs and care in both cases. Indeed, mastitis represents the most persistent disease in dairy cows and is associated with financial losses of $19-32 billion per annum (Ryan et al., 2021).

Due to the continued rise in antibiotic resistant strains recovered from infections, combined with the reduced effectiveness of antibiotics, it has become imperative that alternative antimicrobials be developed. Therapeutics with the ability to combat biofilm formation by interfering with quorum sensing processes or by inhibiting absorption offer substitute modes of actions to traditional antimicrobials. The use of dual-antimicrobials with differing modes of action as a means of overcoming bacterial resistance is an attractive idea, taking advantage of the synergistic effects that these antimicrobials have on each other (Field et al., 2016). Such antimicrobials include the lantibiotic class of bacteriocins, bacteriophages and their lysins. The aim of this review is to provide an updated evaluation of the bacteriocins, phages and phage endolysins that show potential to combat MRSA in the nosocomial environment.

## Genetics and Virulence of Methicillin-Resistant *S. aureus*

**FIGURE 1:**
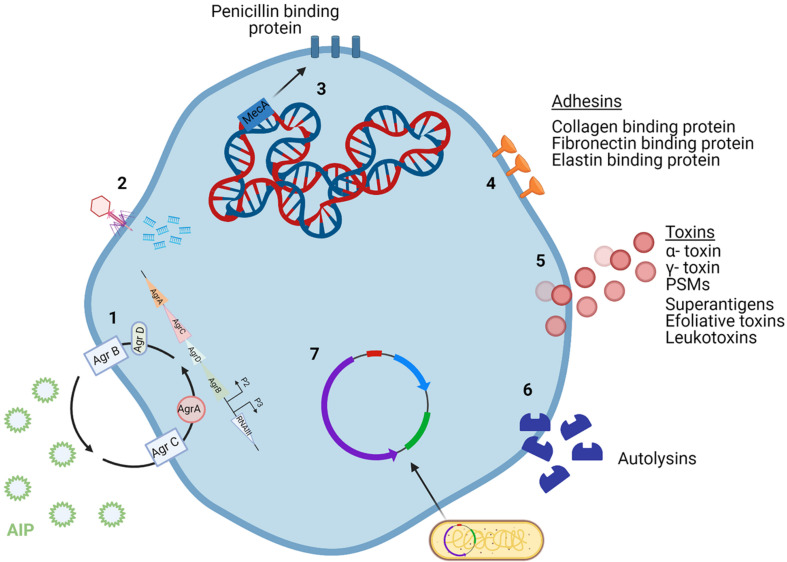
*Staphylococcus aureus* mechanisms of virulence. (1) The *agr* quorum sensing system uses AIP levels to regulate cell wall proteins. (2) Phages incorporate their DNA into the bacterial cell genome. (3) The core genome of *S. aureus* contains a variety of virulence genes, including *mecA* which encodes for methicillin resistance. (4) Adhesins are surface proteins used to bind to host tissue. (5) Toxins secreted by the bacterial cell damage host tissue and can be encoded by the core genome or MGEs. (6) Autolysins break down peptidoglycan in the cell wall and so can attack other cells. (7) Plasmids carry virulence and antibiotic resistance genes that can be incorporated into the genome of bacterial cells. Created using Biorender.com.

The virulence and antibiotic resistance traits of *S. aureus* are governed by genes present on its circular chromosome and extrachromosomal components. These genes can be easily transferred between different bacteria, be it other staphylococcal strains or other Gram positive bacteria (Lowy, 1998). Despite this propensity for genetic exchange, *S. aureus* comprises a conserved genome, lending to the notion that *S. aureus* is a clonal species (Feil et al., 2003). In terms of diversity amongst this species, mobile genetic elements (MGEs) are present which include a variety of prophages and phage-related genomic islands, including *S. aureus* pathogenicity islands (SaPIs), staphylococcal chromosome cassettes (SCCs), transposons and plasmids (Figure 1; Lindsay, 2010; Lindsay et al., 2012). The prophages supply the bacteria with virulence factors necessary for a pathogenic *S. aureus* strain to effectively infect a mammalian host cell (Brüssow et al., 2004; Lindsay, 2010). MRSA strains carry a *mecA* gene that encodes a penicillin–binding protein that confers methicillin resistance (Enright et al., 2002). Methicillin resistance in *S. aureus* strains can also be mediated by the presence of a *mecC* gene, however, this is a less likely occurrence (Newstead et al., 2020). This gene is found on the staphylococcal cassette chromosome, *mec*, which is also a mobile genetic element (Enright et al., 2002). While there are five main SCCmec types (I-V), types I-III are largely found to be the cause of nosocomial *S. aureus* infection (Collins et al., 2010). The evolution of community acquired (CA) MRSA has been considered to be a result of increased virulence in MRSA strains. These strains have incorporated MGEs containing enhanced virulence genes into their genome. This allowed for MRSA strains, which previously had only been found in the nosocomial environment, to occupy a new niche and infect healthy individuals in the community (Otto, 2012).

**FIGURE 2:**
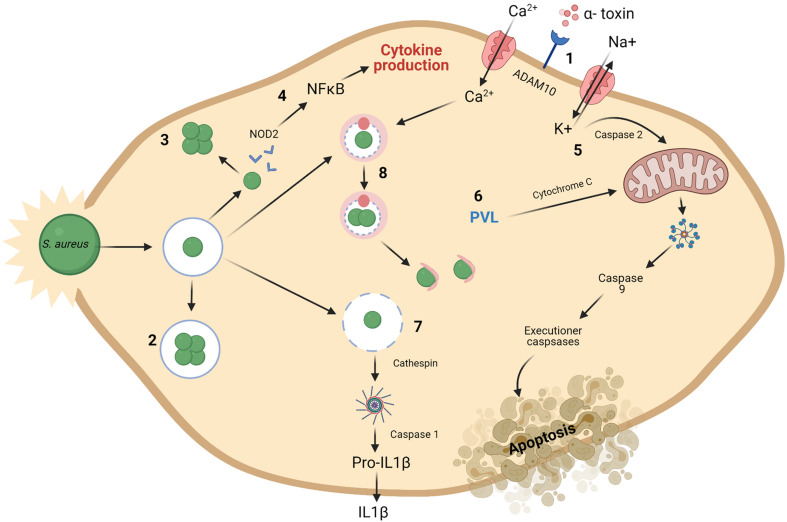
Mechanisms for intracellular preservation of *S. aureus* and induction of cell death. (1) The ADAM10 receptor is a target for formation of α-toxin pores on the cell membrane. (2) *S. aureus* may survive and grow once engulfed in endosomes. (3) or in the cytoplasm of the cell. (4) *S. aureus* present in the cytoplasm are recognized by NOD2 and subsequent activation of NFκB occurs resulting in the production of cytokines. Mitochondrial permeabilization may result in apoptosis. (5) This is brought about by a potassium efflux caused by α-toxin build up and subsequent caspase 2 activity. (6) The presence of PVL also cause disruptions to the mitochondrial membrane. This leads to chain of activation involving cytochrome C, caspase 9 and executioner caspases resulting in apoptosis of the cell. (7) Damaged phagosomes release cathepsin, activating the inflammasome and caspase 1. This activation results in cellular secretion of IL1β, followed by cell death. A process known as pyronecrosis. (8) Bacteria can replicate within autophagosomes, which have collected cellular contaminants, until they can escape at which point they can induce cell death. Macroautophagy is also said to be activated by Ca^2 +^ (Fraunholz and Sinha, 2012). Created using Biorender.com.

The genes responsible for *S. aureus* infection fall under three categories: immunomodulators, adhesins, and toxins. Immunomodulators are proteins that prevent the onset of the host immune response (Figure 2). Adhesins are proteins attached to the surface of the cell, allowing for the bacterium to attach to various tissues of the host (Figure 1). Toxins are proteins that are secreted by the cell to cause damage to the host tissue (Figure 1; Collins et al., 2010). Toxins such as α- toxin, γ- toxin and phenol soluble modulins (PSMs) are encoded by the core genome of most *S. aureus* strains. PSM-mec is an exception in that it is a PSM that is encoded by MGEs. PSMs are amphipathic peptides that have demonstrated cytolytic capabilities toward neutrophils (Otto, 2012). MGEs encode for a variety of other toxins including superantigens, exfoliative toxins and leukotoxins (Chatterjee et al., 1992; Queck et al., 2009). Panton-Valentine leucocidin (PVL) is a pore-forming bi-compartmental toxin. Initially, it was believed that the presence of PVL genes in the genome of MRSA strains led to the increased virulence of those strains. This was because PVL genes *lukS* and *lukF* were found predominantly in CA-MRSA, with very little evidence for their presence in hospital acquired (HA) MRSA (Vandenesch et al., 2003). However, evidence has since emerged showing that PVL genes have been found in HA-MRSA strains (Otto, 2012). Bacterial autolysins are enzymes produced by the cell to accommodate cell division. Autolysins are encoded by *atl* and are capable of breaking down peptidoglycan in the cell wall by means of their glucosaminidase and amidase activities (Haghighat et al., 2017). The physiology of *S. aureus* is controlled by multiple regulatory systems, including *agr* (accessory gene regulator) system. The *agr* system is an important regulator for the virulence capabilities of *S. aureus*. This quorum sensing system operates by monitoring the production of the autoinducing peptide (AIP) and regulating cell wall-associated protein expression accordingly (Traber et al., 2008).

Small-colony variants (SCVs) are bacterial variants that have lower metabolic activity than standard *S. aureus* cells and are also more readily able to resist the action of antibiotics (De Maesschalck et al., 2020). Their ability to resist antibiotics comes from their reduced metabolic rate and slow growing phenotype (Leimer et al., 2016). There is growing support for the relevance of SCVs in a clinical setting as evidence continues to emerge that they are contributing to the development of antibiotic resistance (Peyrusson et al., 2020). From a clinical perspective, there is little known on how induction of SCVs in patients arises (Leimer et al., 2016). The ability of *S. aureus* to persist inside eukaryotic cells is a driving force behind chronic *S. aureus* infections (Röhrig et al., 2020). *S. aureus* intracellular persistence is achieved through various mechanisms, as demonstrated in Figure 2. Bacterial cells replicate either in the cell cytoplasm having escaped the phagosome, or within the phagosome itself. Once intracellular, *S. aureus* cells have successfully evaded eradication and induced cell death through pyronecrosis and apoptosis (Fraunholz and Sinha, 2012). Staphylococcal toxins, such as PVL, can trigger pyroptosis in keratinocytes, resulting from activation of the inflammasome induced by caspase 1 (Soong et al., 2015).

*Staphylococcus aureus* is commonly recognized as one of the most significant clinical pathogens in terms of biofilm formation capacity on implanted medical devices (Kelly et al., 2012). The ability of a bacterial species to form biofilms significantly increases its likelihood of withstanding antimicrobial attack. In contrast to planktonic cells, the bacterial cells present in a biofilm are physiologically and physically protected from antibiotics by sequential layers of growing microbes (de la Fuente-Núñez et al., 2013). While the exterior layers of the biofilm may be killed on exposure, the interior layers remain untouched and so can re-establish the bacterial population which then reforms the biofilm once the antimicrobial effects have subsided. The ability of *S. aureus* to form biofilms and colonize efficiently is aided by surface proteins including SasX (Figueiredo et al., 2017).

## Bacteriocins

### Background

**FIGURE 3:**
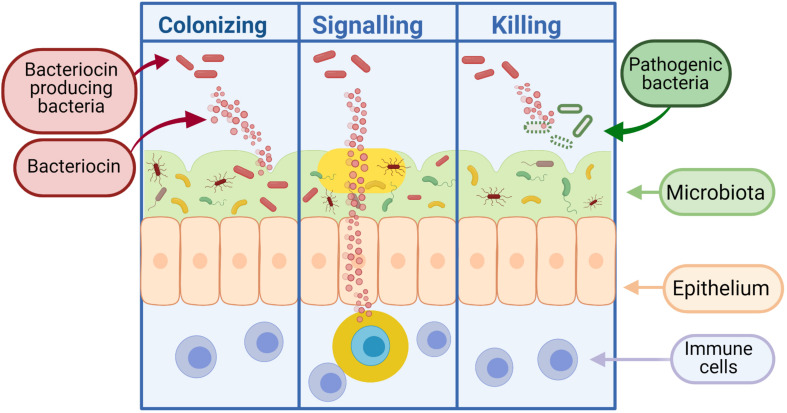
Bacteriocins can function as colonizing peptides (by opening up a niche in the environment for their producing bacteria to occupy), as signaling peptides [to communicate with the immune system or other surrounding bacteria, or as killing peptides (to eradicate bacteria threatening the survival of their producing bacteria)] (Dobson et al., 2012). Created using Biorender.com.

Bacteria can produce antimicrobial peptides, known as bacteriocins, which provide defense against other closely related strains (narrow spectrum) or unrelated species (broad spectrum). Bacteriocins were first discovered in Gratia (1925) and have been the focus of much research due to their bactericidal nature and ability to inhibit the growth of pathogenic bacteria. It is thought that the majority of archaea and bacteria produce one if not more bacteriocins (Klaenhammer, 1988). Although the process of producing bacteriocins has been shown to be quite energy demanding on the cell, the staggering amount of bacteria that produce bacteriocins supports the idea that this activity is central to the competitiveness of bacteria in some way, be it through killing and/or communication (Gillor et al., 2009). While early applications for bacteriocins were primarily based on food preservation, recent repurposing of these peptides has led to the development of therapeutics for human health. As shown in Figure 3, bacteriocins act as signaling peptides to communicate with surrounding communities of microbes, as well as facilitating communication with mammalian immune cells; colonizing peptides allowing for the growth of their producing bacteria and enabling cells to occupy a niche in a competitive environment (Riley and Wertz, 2002); or killing peptides which act to remove competing bacteria from the environment.

**FIGURE 4:**
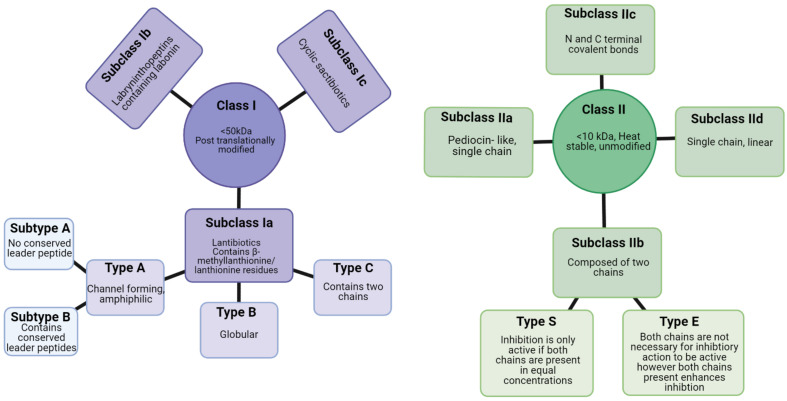
Class I and class II bacteriocins produced from Gram positive bacteria (Newstead et al., 2020). Created using Biorender.com.

Bacteriocins can be divided into two classes: Class I, lanthioine-containing bacteriocins (also referred to as lantibiotics), and Class II, non-lanthionine-containing bacteriocins (Cotter et al., 2005; Figure 4). In Class I lantibiotics, post-translational modifications result in the formation of the unique amino acids, b-methyllanthionine and lanthionine. There has been rapid growth in the identification of new lantibiotics – so it comes as no surprise that they are gaining traction in the search for new antimicrobials. Some lantibiotics, such as lacticin 3147, are composed of two peptides, while nisin is composed of a single peptide. A key component in the biosynthesis of peptidoglycan is lipid II, which many lantibiotics such as nisin have the ability to bind. This binding process is generally followed by the formation of a pore in the target bacterial cell (Piper et al., 2009). The N- terminus present in the hinge region of the peptide is critical to this binding process (Healy et al., 2013). The N- terminus is composed of three (b-methyl) lanthionine rings, termed rings A, B, and C (incorporated at post-translational level). The N- and C- termini are linked by a hinge region (Field et al., 2008). The C- terminus contains two rings of its own, termed rings D and E. The C terminus is responsible for the antimicrobial activity which allows for pore formation in the bacterial cell (Healy et al., 2013). This results in the flow of ions and cytoplasmic components out of the target cell leading to extreme damage and subsequent cell death (Field et al., 2008). The bacteriocin producer is generally resistant to its own bacteriocin due to the presence of immunity gene(s) within the bacteriocin gene cluster in addition to the production of immunity protein(s).

As a potential basis for new antimicrobial therapies, bacteriocins have several advantages over conventional antibiotic treatments. Bacteriocins generally have narrow to broad inhibition spectra – which means that insensitive commensal bacteria in microbiomes remain unperturbed. In the gut, for example, disruptions to the surrounding microbiota as a consequence of broad-spectrum antibiotic treatment can lead to further complications and enable pathogenic bacteria to flourish. A specific example of this is *Clostridioides difficile* infections which can flourish in the gut following eradication of commensals via antibiotic use and can cause mild to fatal diarrhea (Buffie et al., 2012). The mode of action for bacteriocins is generally different from that of specific antibiotics, allowing bacteriocins to kill bacterial strains which are resistant to antibiotics (Cotter et al., 2013). Furthermore, the concentrations at which bacteriocins exhibit antimicrobial activity are far less than those required by antibiotics (Newstead et al., 2020). Finally, bacteriocins lend themselves to the production of derivatives through peptide bioengineering (Cotter et al., 2013; Field et al., 2015). In this way, variants with enhanced and desirable characteristics can be produced, yielding bacteriocins that are more “fit for purpose” in terms of therapeutic applications. For example, Field et al. (2008) has generated variants of nisin, N20P, K22S and M21V which exhibit increased antimicrobial activity to a variety of pathogens including various staphylococci.

### Bacteriocins Utilizing Nanotechnology

Delivery systems have been developed to minimize degradation of bacteriocins by gastric enzymes and maximize therapeutic effect. The use of nanotechnology to enhance characteristics such as antimicrobial activity, bioavailability, short half-life and delivery potential of bacteriocins is a relatively new area of research. Nanotechnology is the use of 1-100 nm-sized particles known as nanoparticles that are made of carbon, metal or organic matter (Sulthana and Archer, 2020). Research on lipid-based nanoparticles has led to the development of nanoliposomes and solid lipid nanoparticles (SLN) (Banerjee, 2001; da Silva Malheiros et al., 2012). Phytogylogen nanoparticles and chitosan/alginate nanoparticles fall under the category of carbohydrate-based nanoparticles (Fahim et al., 2016). Many studies have shown how nanoparticles can improve the capabilities of bacteriocins. A chitosan/alginate combination was used to encapsulate nisin and resulted in increased activity against the *S. aureus* ATCC 19117 strain in ultra-filtered feta cheese (Zohri et al., 2013). An injectable polysaccharide gel has been shown to effectively encapsulate and release nisin to treat *S. aureus* infections and maintain antimicrobial activity for up to 10 days (Flynn et al., 2020). The composition of the gel included oxidized dextran, glycol chitosan and alginate which was activated by the addition of hydrazine groups. Researchers varied the incorporation of gel components which allowed for manipulation of the swelling capabilities of the gel and the subsequent release of nisin. Conjugation with nanosized gold or silver particles increases binding to the negatively charged bacterial membrane due to the positive charge on the nanoparticles. Oxidative stress is then induced in the bacterial cell and subsequent cell death occurs (Seil and Webster, 2012). Incorporation of bacteriocins into polymeric nanofibers is achieved by electrospinning the bacteriocin (Fahim et al., 2016). In particular, this technique has enhanced bacteriocins to treat *S. aureus* and MRSA infection *in vitro and in vivo*. Heunis et al. (2013) developed a nisin nanofiber wound dressing which reduced the bacterial load of skin wounds on mice by five logs after 7 days of treatment when compared with the control (Heunis et al., 2013). Another study examining the effect of nisin-incorporated nanofibers on a biofilm-forming MRSA strain found that the treatment reduced biofilm formation by 88% (Ahire and Dicks, 2015) Although the use of nanotechnology to develop bacteriocin treatment is showing potential, much is still unknown, including if and what potential interactions take place between the nanomaterials, peptides and target bacteria. More studies are required to determine the impact this technology has on the antimicrobial activity of the bacteriocin. For clinical studies, toxicity risks and pharmacokinetics data need to be established.

### Limitations of Bacteriocins

Although treatments utilizing bacteriocins have various advantages over antibiotics, this form of therapy also has numerous shortcomings. While in some respects the narrow activity spectrum of most bacteriocins has its benefits, as previously mentioned, this can also be seen as a negative. Indeed, it is essential that the infection-causing microbe(s) are known for treatment to be successful, rendering administration of the treatment slightly more time consuming than that of antibiotic therapy as the causative agent(s) is identified. When considering the use of orally administered therapeutics, it is important to consider factors such as bioavailability, half-life and absorption achieved by the intestine. An issue contributing to the challenge of using bacteriocins as therapeutics is their inability to survive the conditions of the gastrointestinal tract. This is a result of peptide degradation by digestive enzymes and changes in pH, and has been demonstrated in various *in vitro* and *in vivo* studies (Heinemann and Williams, 1966; Jarvis and Mahoney, 1969; Newstead et al., 2020). The sensitivity of most bacteriocins to gut proteases dramatically decreases their half-life in comparison to antibiotics (Hols et al., 2019). One study examined the ability of the two-component, anti-*C. difficile* bacteriocin, thuricin CD, to survive gastric enzymes and the subsequent bioavailability of the bacteriocin. The Trnβ peptide of the bacteriocin was degraded by α- chymotrypsin and pepsin (Rea et al., 2014). Some studies have shown that these issues can be overcome through peptide bioengineering. Specifically, researchers showed that proteolytic cleavage of a chemically synthesized peptide was reduced following the addition of d-amino acids (Hoskin and Ramamoorthy, 2008). Parenteral administration of bacteriocins will circumvent exposure of peptides to gut proteases, however, some protease interaction still takes place due to fibrinolysis and hemostasis occurring in the bloodstream (Mathur et al., 2017). Furthermore, some bacteriocins have exhibited immunogenic activity which could compromise their use for systemic infections (Soltani et al., 2021). While bacteriocins are generally considered non-toxic, studies have shown that at high concentrations bacteriocins show signs of cytotoxicity toward various cell lines (Chalekson et al., 2003; Soltani et al., 2021). A limited number of studies have examined the toxicity of bacteriocins *in vivo* (Soltani et al., 2021). One study investigated the immunogenicity and toxicity of the peptide P34 on BALB/c mice (de Almeida Vaucher et al., 2011). There was no apparent increase in antibody titer or hypersensitivity reaction in the treated group. However, histological changes in the spleen were observed in the group administered P34 (de Almeida Vaucher et al., 2011).

The methods by which purified bacteriocins are produced are less than satisfactory due to slow processes and high costs. Traditionally, purified bacteriocin is produced by batch fermentation with the producer strain. This strategy requires specific components depending on which bacteriocin is being produced, creating difficulties in mass producing a variety of bacteriocins (Hols et al., 2019).

Bacterial exposure to even low concentrations of bacteriocin may result in resistance (Van Schaik et al., 1999). This cellular response is a means of adapting to and surviving the changing conditions in the environment. Resistance is achieved via alterations to structural components such as the cell wall and membrane of the bacterial cell. These alterations can affect cellular regulation with regard to membrane lipid composition, electrical fluidity and cell wall density (Mantovani and Russell, 2001). Lantibiotics interact with lipid II contained in the cytoplasmic membrane and subsequently create a pore. In addition to that, some lantibiotics, such as nisin, prevent the process of binding with lipid II, which in turn stops the synthesis of the cell wall. In contrast to that, the interaction of two-component lantibiotics with the cytoplasmic membrane is mediated by the A1 peptide. Once this interaction has been established, lipid II is then targeted (Wiedemann et al., 2006). Class II bacteriocins use the mannose-phosphotransferase system to interact with the cell membrane and cause subsequent permeability of the membrane (Nissen-Meyer et al., 2009). *L. monocytogenes* has demonstrated resistance to bacteriocins pediocin PA-1, nisin and leuconocin S (Bruno and Montville, 1993) Another example of bacterial resistance was observed in *Bacillus cereus* and *Paenibacillus polymyxa* which produce the degradation enzyme, nisinase, capable of degrading nisin (Meade et al., 2020). Only limited knowledge exists on *S. aureus* resistance to bacteriocins. Due to the restricted number of *in vivo* studies examining bacteriocin resistance, the majority of knowledge in this area is derived from *in vitro* studies. While this information gives an insight into how bacteriocin resistance may occur in a clinical setting, further studies are necessary to fully understand the threat of bacterial resistance in such a setting. However, nanotechnology and combinatorial therapies should help to overcome bacterial resistance to bacteriocins.

### *In vitro* and *in vivo* Experiments of Bacteriocins Against MRSA

*Staphylococcus capitis* APC2923 isolated from the epithelial surface layer of the toe web space is known to produce a bacteriocin termed nisin J (O’Sullivan et al., 2019). This bacteriocin is a nisin variant that has shown activity against clinically significant gram-positive pathogens such as MRSA. Nisin J exhibited increased antimicrobial activity on 12 out of 13 strains tested when compared to nisin A and nisin Z *in vitro*. Gallidermin is a peptide produced by *Staphylococcus gallinarum* that has lytic activity against both methicillin sensitive *S. aureus* and MRSA strains (Manosroi et al., 2010; Bengtsson et al., 2018). The cytotoxic and pro-inflammatory effects of gallidermin on fibroblasts were examined *in vitro*. Only high levels of gallidermin invoked a minor response from CXCL8 and interleukin-6. The bacteriocin was able to suppress an inflammatory response caused by the *S. aureus* infection (Bengtsson et al., 2018). While gallidermin is capable of suppressing the growth of *S. aureus* biofilms, the ability of the bacteriocin to eradicate a biofilm that has already formed is less impressive (Saising et al., 2012). Similarly, hyicin 4244 has shown an ability to inhibit MRSA strains and reduce an *S. aureus* biofilm. This bacteriocin is produced by *Staphylococcus hyicus* 4244 and classed as a sactibiotic (de Souza Duarte et al., 2018). Hyicin was examined, *in vitro*, for its ability to inhibit mastitis-causing *S. aureus* isolates. All isolates tested were sensitive to hyicin activity. Furthermore, hyicin showed impressive ability at biofilm eradication, reducing the number of viable cells by 99.9%. BacSp222 has also demonstrated an ability to inhibit various *S. aureus* strains, including MRSA. This bacteriocin, produced by *Staphylococcus pseudintermedius*, is a class II staphylococcin. BacSp222 was active against 15 strains *in vitro*, however, it exhibited cytotoxic effects toward eukaryotic cells (Wladyka et al., 2015). Jang et al. (2020) have isolated a cytoplasm-bound thermolabile bacteriocin from *S. epidermidis* which appears to be highly active against *S. aureus* and MRSA strains *in vitro*. Liu et al. (2011) developed a probiotic capable of producing lysostaphin with the aim of reducing toxic shock syndrome toxin (TSST)-producing *S. aureus*. Elevated pH, carbon dioxide, oxygen and protein levels in the vaginal tract creates an ideal environment for *S. aureus* growth and subsequent production of TSST (Beller and Schweppe, 1979; McCormick et al., 2001). A plasmid containing genes for lysostaphin expression, a promoter responsible for response under neutral pH conditions and a signal sequence was transformed into the probiotic strain *Lactobacillus plantarum* WCFS1. This lysin-modified probiotic produced zones of clearing against an *S. aureus* strain, which produces TSST, at a neutral pH *in vitro* (Liu et al., 2011).

Studies have been carried out utilizing dual antimicrobial actions (bacteriocins and antibiotics combined) for treating *S. aureus* infections. *Lactococcus garvieae* produces a bacteriocin, termed garvicin KS, which is composed of three peptides. Garvicin KS has a broad spectrum of inhibition against Gram positive bacteria as well as some Gram negative bacteria from the genus *Acinetobacter* (Chi, 2018). When tested against a variety of *S. aureus* strains, garvicin KS was active against all strains but three. Using a checkerboard assay garvicin KS was shown to successfully inhibit *S. aureus* growth in conjunction with nisin and farnesol. This synergistic combination reduced the minimum inhibitory concentration (MIC) of garvicin KS by a factor of 8 (Chi and Holo, 2018). Enterocins DD28 and DD93, Class IIb bacteriocins produced by *E. faecalis* 28 and *E. faecalis* 93, respectively, have also demonstrated activity against various *S. aureus* strains, including MRSA. They have exhibited a synergistic effect when used in combination with antibiotics such as kanamycin and erythromycin, resulting in increased activity against MRSA strains. Enterocins DD28 and DD93 are capable of effectively preventing MRSA biofilm growth as well as blocking initial biofilm establishment. This was demonstrated by a 100-fold reduction of MRSA-S1 biofilm cell numbers following treatment with Enterocins DD28 and DD93 in combination with kanamycin and erythromycin (Al Atya et al., 2016). Ellis et al. (2019) utilized sub-inhibitory concentrations of lacticin3147 to promote the dual effect of penicillin and vancomycin, resulting in the inhibition of various bacterial strains including MRSA. Similarly, this study examined the effects of nisin in combination with methicillin, which also produced impressive anti-MRSA activity (Ellis et al., 2019).

Various *in vivo* experiments have been performed to determine the effects of bacteriocins against numerous MRSA associated infections, as well as the effects on the host. Sublancin is a bacteriocin produced by *Bacillus subtilis* 168 and was examined for its anti-MRSA activity in a mouse model. The mice were intraperitoneally infected with MRSA ATCC43300. Sublancin was shown to reduce the bacterial load of the intraperitoneal cavity of the mice. On day 3 of the study, 65% of the control group had died, while an increase in the concentration of sublancin administered correlated to an increase in the survival rate of the mice (Wang et al., 2017). Furthermore, the bacteriocin was able to reduce gut inflammation and equilibrate the immune response of the mice by supressing NFκB activation. Another study examined the effect of a combination therapy, involving garvicin KS, penicillin G and micrococcin P1, on skin infections on a murine model. The strain used in this study was MRSA Xen31, which is a luciferase-tagged strain allowing for observation of fluorescence to represent live cells. The antimicrobial combination treatment was applied in a hydroxypropyl cellulose-based solution. Following infection with MRSA Xen31, the bacteriocin/antibiotic combination was able to eradicate the infection from the wound, while also out-performing a commonly used antibiotic treatment for skin infection, Fucidin cream (Ovchinnikov et al., 2020).

### Clinical Uses of Bacteriocins Against MRSA

Bacteriocins are being used successfully in the food industry to control and inhibit bacterial growth, indeed, nisin was first introduced as a food preservative (E324) in 1969. In veterinary medicine, bacteriocins have been proven to be as effective as leading antibiotics for treating and preventing bovine mastitis (Cao et al., 2007; Wu et al., 2007; Klostermann et al., 2010; Kitching et al., 2019). Although nisin had previously been examined for its activity against *Mycobacterium tuberculosis* (Bavin et al., 1952), its application to combat infections in humans is largely unexplored. In general, studies and clinical trials investigating the efficacy of bacteriocins in humans are limited, with very few addressing *S. aureus* infection. One study that did investigate the ability of nisin to treat human mastitis, which is commonly caused by *S. aureus*, was performed by Fernández et al. (2008). The study was carried out on eight women divided evenly into a treatment and a control group. A nisin solution was applied to the nipple and mammary areola of women in the treatment group, with the control group receiving a placebo solution. Following two weeks of the study, the women in the treatment group had on average 2-logs less of a bacterial load compared to the control group. There were no clinical signs of mastitis in the treatment group following two weeks of treatment with nisin; however, mastitis in the control group remained for the entirety of the study. Nisin is currently being investigated in a clinical trial examining its effectiveness at treating infections causing ventilator-associated pneumonia, and one of the pathogens of interest in this trial is *S. aureus* (Gradisteanu Pircalabioru et al., 2021). Intrabiotics is currently performing a phase I clinical trial utilizing nisin and the protegrin-like peptide IB-367 to treat acne (Gradisteanu Pircalabioru et al., 2021).

Many bacteriocins produce impressive activity against *S. aureus*, most notably MRSA strains (Table 2). However, there is a lack of knowledge surrounding the full extent of the toxic effects that these peptides impart on human hosts. Yet due to the nature of MRSA infections and the degree to which such infections remain a problem in the nosocomial environment, further research into bacteriocin toxicity versus their anti-MRSA efficacy is warranted (Newstead et al., 2020).

**TABLE 2:** List of Bacteriocins active against *S. aureus*.

Bacteriocin	Producing strain	Class	*S. aureus* target strains	Inhibitory potential (MIC)	Pros and cons	References
Hominicin	*Staphylococcus hominis* MBBL 2-9	Ia A1	*S. aureus* ATCC 25923 *S. aureus* (MRSA) ATCC 11435 *S. aureus* (VISA) CCARM 3501	0.06 μg/mL 0.96 μg/mL 3.82 μg/mL		Sung et al., 2010
Nisin J	*Staphylococcus capitis* APC 2923	Ia A1	*S. aureus* DPC 7016 MRSA DPC 5645	*ND		O’Sullivan et al., 2019
Pumilicin 4	*Bacillus pumilus*		MRSA 1297 MRSA 1302 MRSA 1304	*ND	Pro: Active against VRE strains	Aunpad and Na-Bangchang, 2007
Epidermin	*Staphylococcus epidermidis*	Ia A1	*S. aureus* SG 511 *S. aureus* E88	8 μg/mL 8 μg/mL		Kellner et al., 1988; Schnell et al., 1988; Bonelli et al., 2006
Pep5	*Staphylococcus epidermidis*	Ia A1	*S. aureus* FPR3757 (MRSA) *S. aureus* USA300 (MRSA) *S. aureus* MW2 (MRSA) *S. aureus* MSSA252 *S. aureus* Newman	13.15 μg/mL 13.15 μg/mL 23 μg/mL 9.86 μg/mL 13.15 μg/mL		Oppedijk, 2017
Gallidermin	*Staphylococcus gallinarum*	Ia A1	*S. aureus* SG 511 *S. aureus* E88 MRSA	4 μg/mL 8 μg/mL	Pro: Anti-staphylococcal biofilm activity	Kellner et al., 1988; Kempf et al., 2001; Bonelli et al., 2006; Götz et al., 2014
Hyicin 4244	*Staphylococcus hyicus* 4244	Ic	*S. aureus* 4124	*ND	Pro:Anti-staphylococcal biofilm activity Pro: Active against CoNS	de Souza Duarte et al., 2018
BacSp222	*Staphylococcus pseudintermedius* 222	IId	MRSA USA300 strain FPR3757 *S. aureus* KB/8658 *S. aureus* ATCC 25923 *S. aureus* DSM 26258 (CH91)	5 μg/mL 7.7 μg/mL 5.9 μg/mL 5.44 μg/mL	Con: Significant cytotoxic activities toward eukaryotic cells	Wladyka et al., 2015; Pieta et al., 2016
Garvicin KS	*Lactococcus garvieae KS1546*	IIe	*S. aureus* LMGT 3242	*ND	Pro: Broad spectrum of inhibition	Chi and Holo, 2018
Epidermicin NI01	*Staphylococcus epidermidis* 224	IId	MRSA s37 MRSA s41 MRSA s71 *S. aureus* 1195	1-2 μg/mL 1-2 μg/mL 2 μg/mL 2 μg/mL	Pro: Anti-staphylococcal biofilm activity	Sandiford and Upton, 2012
Bactofencin A	*Lactobacillus salivarius* DPC6502	IId	*S. aureus* 5246	2.7-13.9 μg/mL		O’Shea et al., 2013
Lysostaphin	*Staphylococcus simulans*	IIIa	MRSA 301	0.0625 μg/mL	Pro: *In vitro* and *in vivo* experiments	Schindler and Schuhardt, 1965; Dajcs et al., 2000
Epilancin 15X	*Staphylococcus epidermidis* 15 × 154	Ia A1	Multi drug resistant *S. aureus*	*ND		Ekkelenkamp et al., 2005; Velásquez et al., 2011
Hyicin3682	*Staphylococcus hyicus* 3682	Ia A1	*S. aureus* 2S2 *S. aureus* 4S1	*ND		Fagundes et al., 2011
C55	*Staphylococcus aureus* C55	IIb S	MRSA		Pro: Active against mupirocin resistant *S. aureus* strains	Navaratna et al., 1998
Aureocin A53	*Staphylococcus aureus* A53	IId	*S. aureus* ATCC 29213	769.6 μg/mL		Netz et al., 2002; Acedo et al., 2016
Endopeptidase ALE-1	*Staphylococcus capitis* EPk1	IIIa	*S. aureus* FDA209P	54.2 μg/mL		Sugai et al., 1997
Nisin M21V	*Lactococcus lactis* NZ9800	Ia A1	*S. aureus* ST 528 (MRSA) *S. aureus* ST 530 (MRSA) *S. aureus* ST 534 (MRSA)	0.26 μg/mL 0.26 μg/mL 0.52 μg/mL	Pro: Anti-staphylococcal biofilm activity	Field et al., 2008, 2010, 2016
Nisin A	*Lactococcus lactis* NZ9800	Ia A1	*S. aureus* MR23 (MRSA)	8.4 μg/mL	Pro: Anti-staphylococcal biofilm activity	Field et al., 2008; Okuda et al., 2013
Sublancin	*Bacillus subtilis168*	Ia	*S. aureus* MRSA ATCC43300	>100 μg/mL 15 μM		Paik et al., 1998; Wang et al., 2017
Lacticin Q	*Lactococcus lactis* QU 5	IId	*S. aureus* MR23 (MRSA)	29.6 μg/mL	Pro: Anti-staphylococcal biofilm activity	Okuda et al., 2013
BAC-IB17	*Bacillus subtilis KIBGE-IB17*	*ND	MRSA	50 μg/mL		Ansari et al., 2018
Mersacidin	*Bacillus* sp. strain HIL Y-85,54728	IIa	*S. aureus* 99308 (MRSA)	1 μg/mL	Pro: *In vitro* and *in vivo* experiments	Kruszewska et al., 2004
Enterocin DD28	*Enterococcus faecalis* 28	IIb	MRSA ATCC 43300 MRSA-S1 MRSA-2 *S. aureus* ATCC33862	200 μg/mL 200 μg/mL 200 μg/mL 100 μg/mL	Pro: Anti- staphylococcal biofilm activity	Al Atya et al., 2016
Enterocin DD93	*Enterococcus faecalis* 93	IIb	MRSA ATCC 43300 MRSA-S1 MRSA-2 *S. aureus* ATCC33862	200 μg/mL 200 μg/mL 200 μg/mL 100 μg/mL	Pro: Anti- staphylococcal biofilm activity	Al Atya et al., 2016
TA6	*Pseudomonas aeruginosa* TA6	II	MRSA	*ND		Arumugam et al., 2019
Pentocin JL-1	*Lactobacillus pentosus*	I	MRSA GIM 1.771	7.5 μg/mL	Pro: Active against gram positive and gram negative bacteria Active against multi drug resistant bacteria	Jiang et al., 2017
DS-3	*Brevibacillus latersporus*	*ND	MRSA *S. aureus* ATCC29212 *S. aureus* ATCC 25923	*ND		Odah et al., 2019

*ND, not disclosed.

## Phages

### Background

Phages are viruses that infect bacteria and may enter either the lytic or lysogenic life cycle following infection. During the lytic lifecycle, infecting phages undergo phage replication and assembly followed by release from the infected cell subsequently resulting in cell death. More specifically, following the assembly and packaging of phage particles in the infected cell, enzymes known as endolysins are produced by the phage to lyse the bacterial host from within. During the lysogenic lifecycle, the phage genome integrates into the bacterial host genome or may be present in the cell in the form of a plasmid. The phage genome then replicates with the host genome and is referred to as a prophage (Salmond and Fineran, 2015). The lysogenic phage/prophage essentially remains dormant until the host cell is exposed to a stressor such as nutrient limitation, in which case the prophage is induced to lyse the host cell. Phages can be solely lytic, generally referred to as lytic phages, or can have the capacity to enter lysogeny, referred to as lysogenic phages.

**FIGURE 5:**
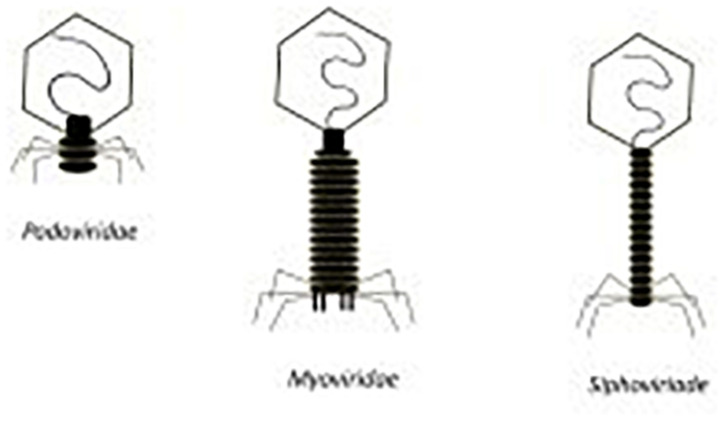
Morphology of *podoviridae, myoviridae*, and *siphoviridae* phages (Newstead et al., 2020).

Phages may have single-stranded or double-stranded RNA or DNA genomes housed in the capsid head of the phage. The Caudovirales order of phages have double-stranded linear DNA genomes and consist of the *Podoviridae*, *Siphoviridae* and *Myoviridae* families of tailed phages (See Figure 5) (Ackermann, 2011). The families of *Siphoviridae* and *Myoviridae* represent most of the lytic phage community (Ackermann, 2011). *Myoviridae* phages differ from *Siphoviridae* phages by having a larger capsid head and contractile tails. *Podoviridae* phages have a different morphology with a capsid head that can be isometric, prolate or elongated and short non-contractile tails (Ackermann, 2011). In terms of potential use as an anti-*S. aureus* therapeutic, *Myoviridae* phages have demonstrated an impressive ability to target MRSA cells. Lytic phages from the *Myoviriade* family have been shown to have a broad host range, not only capable of infecting various staphylococcal species but also coagulase-negative species (CoNS) (O’Flaherty et al., 2004; Cui et al., 2012; Melo et al., 2018). Examples of such phages include SA012, K and 812 (Xia et al., 2011; Li et al., 2015; Fujiki et al., 2018).

Phages have been shown to be highly effective in the treatment of some bacterial infections (Wittebole et al., 2014), including infections caused by antibiotic resistant bacteria (Kortright et al., 2019). “Phage therapy” in general describes the use of phages, preferably lytic, to treat bacterial infections. One of the major advantages of phage therapy is that phages are highly specific and have narrow inhibition spectra, thus the target bacterium can be killed while the surrounding microbiota remain untouched. Bacteria are known to harbor phage resistance mechanisms including adsorption inhibition, restriction modification, abortive infection, the CRISPR-Cas system, and BREX, which can disrupt the phage lytic cycle at various stages (Lenski, 1988; Coffey and Ross, 2002; Labrie et al., 2010). Phages can evolve to evade these mechanisms but likewise, bacteria too can evolve to counter attack, resulting in defense and counter-defense cycles. However, the use of phage cocktails (two or more phages) can reduce the likelihood of bacterial resistance development (Gu et al., 2012). Indeed, phage cocktails tend to be the treatment of choice and can be “updated” with specific phages in response to a particular infection, often resulting in personalized treatments. Further to this, it is important to point out that the development of phage resistance in the target bacterium can reduce its fitness and even pathogenicity (León and Bastías, 2015).

**FIGURE 6:**
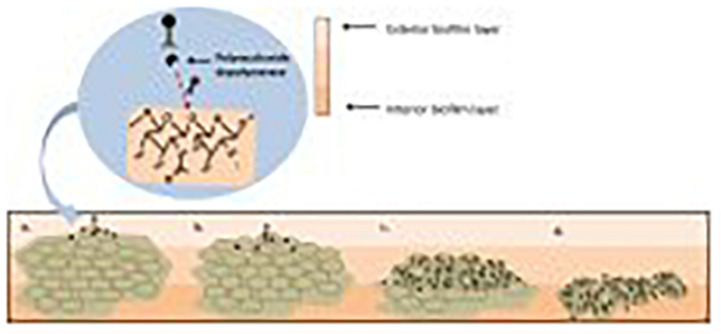
**(a)** Initial biofilm exposure to proteins produced by the phage such as polysaccharide depolymerase. **(b)** Breakdown of the extracellular polymer substance allows for phage to infect the bacterial cells. **(c)** Phage infection of the bacterial cells in the exterior layer of the biofilm leads to infection of bacterial cells deeper within the biofilm. **(d)** A combination of the breakdown of the extracellular polymer substance due to polysaccharide depolymerase exposure and phage replication leads to the eradication of the biofilm. (Gutiérrez et al., 2016).

As previously discussed, *S. aureus* strains are capable of efficiently forming biofilms. This creates a significant obstacle when treating *S. aureus* infections, particularly MRSA. Phages have demonstrated an impressive ability to eradicate biofilms when compared with antibiotics (Azeredo and Sutherland, 2008). The cells present in a biofilm are enveloped in an extracellular polymeric substance (Archer et al., 2011). In contrast to antibiotics, some phages can gain access to the bacteria deep within a biofilm due to their production of polysaccharide depolymerase enzymes which break down the extracellular polymeric matrix (de Vries, 2019). Furthermore, phage concentrations increase with the lytic cycle – thus, as phages begin to target exterior layers of the biofilm, more phages are produced that can infect bacterial cells found deeper within the biofilm in a continuous cycle ultimately leading to full eradication as depicted in Figure 6; Clokie et al. (2011).

**FIGURE 7:**
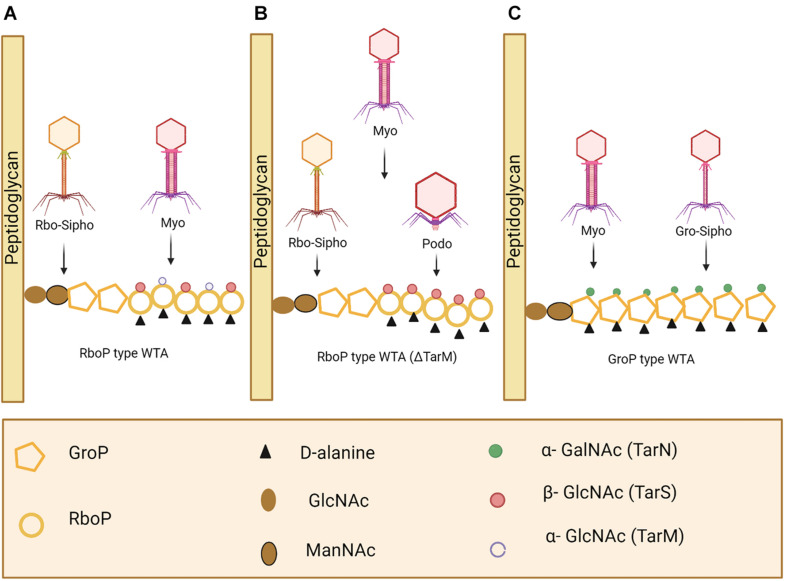
*Staphylococcus aureus* phage recognition of wall teichoic acids (WTA) in *Staphyococcus*. **(A)** Staphylococcal *Myoviridae* (Myo) and *S. aureus Siphoviridae* (Rbo-Sipho) phages can recognize α-GlcNAc and β-GlcNAc residue containing ribitol phosphate (RboP)-type WTA. **(B)** *Podoviridae, Siphoviridae and Myoviridae* phages are capable of infecting *S. aureus* when α-GlcNAc residues are absent. **(C)** Glycerol phosphate (GroP)-type WTA is only recognized by Myoviridae phages and Gro-*Siphoviridae* phages (including CoNS and *S. pseudointermedius* phages) (Azam and Tanji, 2019). Created using Biorender.com.

Phage adsorption to the host cell is the first step in phage infection. Cell wall teichoic acids (WTAs) are glycopolymers that are critical to cell division and are conserved in all *S. aureus* strains (Peng et al., 2019). WTAs can serve as phage receptors on the cell wall of Gram positive bacteria, allowing most *S. aureus* phages to bind (Figure 7; Pollack and Neuhaus, 1994; Meredith et al., 2008). In most cases, WTAs present on *S. aureus* cells are comprised of 1,5-ribitol-phosphate (RboP) repeats with the addition of D-alanine and a GlcNAc residue. The initiation of WTA biosynthesis begins by GlcNAc binding to undecaprenol lipids, a process mediated by TarO, an N-acetylglucosamine transferase. The β-GlcNAc transferase, TarS, and α-GlcNAc transferase, TarM, are responsible for the transfer of α- and β-linked GlcNAc onto WTA (Xia et al., 2010; Brown et al., 2012). WTAs are then transferred to the extracellular surface of the host cell where linking to peptidoglycan (PG) takes place (Estrella et al., 2016). However, some phages take advantage of alternative host cell wall components for mediation of binding. For example, the *S. aureus*-infecting phage *Siphoviridae* SLT uses lipoteichoic acid (LTA) to bind to the host cell and carries the PVL toxin (Kaneko et al., 2009). A recent study reported on the presence of prophages with genes capable of encoding a different WTA glycosyltransferase, in many MRSA strains. TarP mediates the delivery of β-GlcNAc to an alternative RboP WTA hydroxyl group, in comparison to TarS. Due to the necessary presence of β-GlcNAc for phage infection to occur, this alternative route for β-GlcNAc allows for the bacterial cell to avert phage adsorption and subsequently phage infection (Gerlach et al., 2018). Considering the large number of MRSA strains in which this pathway has been observed, using phages that target the β-GlcNAc residue present in WTA no longer seems a logical choice for phage therapy. As depicted in Figure 7, *S. aureus* possesses various phage recognition WTAs.

### Limitations to Phage Therapy

Despite all the aspects of phage therapy that showcase its potential as a therapeutic in human health, there are some limitations. As is the case with bacteriocins, the narrow spectrum of activity of phages is not always beneficial. However, this is overcome by the use of phage cocktails targeting a variety of specific hosts (Royer et al., 2021). Phages may invoke an immune response upon administration which has led to anaphylactic shock or hypersensitivity reactions (Gorski et al., 2012). The ability of phages to transfer genetic material such as virulence or antibiotic resistance genes to the host adds to the problem of antibiotic resistant bacteria (Modi et al., 2013). The lack of uptake in using phage therapy as a frontline therapeutic in western medicine is in part due to the fact that until recently the safety of this form of therapy has been largely unexplored (Lin et al., 2017), but is predominantly due to the difficulty in developing phage therapies that slot into existing regulatory frameworks (Sybesma et al., 2018). Indeed, updating phage cocktails/treatments with suitable phages in response to specific infections is essential if phage therapy is to fully work. However, under current regulatory frameworks, this represents a timely ordeal requiring preclinical, clinical and pharmaceutical documentation. Furthermore, the familiarity that many practitioners have with traditional antibiotic treatment, may pose a threat to the success of phage therapy (Sybesma et al., 2018).

### *In vitro* and *in vivo* Experiments of Phages Against MRSA

Numerous *in vitro* and *in vivo* studies have reported the potential of phages and their lysins to eradicate MRSA infection (as seen in Table 3) (Schmelcher and Loessner, 2021). *In vitro* studies performed on phage Henu2 showed that alone this phage exhibits unimpressive inhibitory action against *S. aureus*. However, in combination with oxides or antibiotics, the phage is capable of a 3-log reduction in bacterial load within 24 h (Li et al., 2021). The ability of a phage to inhibit MRSA infection of skin wounds in a murine model was investigated by Ji et al. (2020). Infection was induced by subcutaneous injection of the MRSA strain, and following 1 h, phage VB_SauS_SHSt was administered. An increased inflammatory response was observed in the untreated group, with the treated group experiencing faster wound rehabilitation and reduced tissue damage (Ji et al., 2020). A rat model was used to determine the efficacy of a recombinant nano phage in treating MRSA ATCC 33591-infected 3^*rd*^ degree wounds. The wounds which received topical application of the phage demonstrated a more efficient healing process (Rahimzadeh et al., 2020). Shetru et al. (2021) also investigated the ability of phage DSMA-2 to reduce *S. aureus* infection of a skin wound. This study examined multiple groups of mice treated with various concentrations of the phage. Phage treatment improved wound healing and tissue remodeling, while also rescuing 100% of the mice from death at a phage concentration of 5 × 10^6^ pfu/mL compared with a 34% survival rate in the untreated group.

**TABLE 3:** Examples of Phage and phage endolysins active against *S. aureus*.

Phage	Lysin	Family	*S. aureus* target	Inhibitory potential	References
	PlySs2		MRSA 8325 MRSA MW2	MIC – 16 μg/mL MIC – 16 μg/mL	Gilmer et al., 2013
	Staphefekt SA.100		Active against 28 MSSA strains and 8 MRSA strains	MIC – 64 μg/mL	Herpers et al., 2016
Phage K		*Myoviridae*	Active on 39 of 53 MRSA isolates tested		O’Flaherty et al., 2005
	LysK		MRSA USA300	MIC – 650nM	Becker et al., 2008
	CHAPk		MRSA 252	MIC - 0.125 μg/mL	Hathaway et al., 2017
	SAL200		MRSA	Treatment protected 95-100% of mice compared to 40-93% of mice that died in the control group	Bae et al., 2019
MH-1		*Myoviridae*	Active on 27 of 30 MRSA isolates tested		Hallajzadeh et al., 2020
MR003		*Myoviridae*	Active on 26 out of 28 MRSA isolates tested		Peng et al., 2019
812		*Myoviridae*	43% of 89 CoNS *S. aureus* isolates tested		Pantůček et al., 1998
Sb-1		*Myoviridae*	Active on 22 of 28 MRSA isolates tested		Tkhilaishvili et al., 2018
ISP		*Myoviridae*	Active on 86% of MRSA isolates tested		Vandersteegen et al., 2011
Stafal (Commercial Preparation of phages)		*Myoviridae*	*S. aureus* SF29 (MRSA) *S. aureus* SF34 (MRSA) *S. aureus* SF92 (MRSA) *S. aureus* SF121 (MRSA) *S. aureus* SF123 (MRSA)	10^8^ PFU/mL of phage eradicated biofilms of all strains following 24 h treatment	Dvořáčková et al., 2019
SCH1		*Podoviridae*	Active on 97% of 31 MRSA isolates tested		Azam and Tanji, 2019
B1		*Myoviridae*	Active on 73.9% of 23 *S. aureus* isolates		Ajuebor et al., 2018
VB_SauS_SH-St		*Siphoviridae*	Active on 32% of MRSA isolates tested		Ji et al., 2020
	Lys109		*S. aureus* CCARM 3090	MIC – 0.375 μM	Son et al., 2021
JA1		*Myoviridae*	Active on 78.2% of 23 *S. aureus* isolates		Ajuebor et al., 2018
Henu2		*Siphoviridae*	Active on 54% of MRSA isolates tested		Li et al., 2021
MR11		*Siphoviridae*	*S. aureus* SA37 MRSA strains MR1, MR13, MR18, or MR28	MOI of 1 protected mice MOI of 50 protected mice up to 7 days after treatment	Matsuzaki et al., 2003
	MV-L		MRSA 85/2082-STR1	MV-L administered directly after (*n* = 10) or 30 minutes (*n* = 10) after infection rescued 100% of mice	Rashel et al., 2007
	GP16		Active on 22 *S. aureus* isolates tested		Rashel et al., 2007
M^*Sa*^			*S. aureus* A170 *S. aureus* A352 (MRSA)	Rescued 97% of Mice (*n* = 5) Rescued 100% of mice (*n* = 5)	Capparelli et al., 2007
SA012		*Myoviridae*	Active on 21 of 28 MRSA isolates		Peng et al., 2019
	Lys-SA012		*S. aureus* 2007-13 (MRSA) *S. aureus* 2007-28 (MRSA) *S. aureus* 2007-57 (MRSA) *S. aureus* 2007-93 (MRSA) *S. aureus* VC39-Vet1 (MRSA) *S. aureus* VC50-Vet1 (MRSA)	Active on all MRSA isolates tested	Fujiki et al., 2018
R4		*Siphoviridae*	Active on 14 MRSA strains tested		Pertics et al., 2020
MR-5		*Myoviridae*	Active against 18/45 clinical MRSA isolates		Kaur et al., 2012
MR-10		*Myoviridae*	*S. aureus* ATCC 43300 (MRSA)	3.5 log reduction on day 3 (*n* = 12)	Chhibber et al., 2013
ME18		*Myoviridae*	*S. aureus* 1S (MRSA) *S. aureus* 8S (MRSA) *S. aureus* 6M (MRSA) *S. aureus* 12M (MRSA)	MOI of 10 significantly reduced biofilm in all strains	Gharieb et al., 2020
ME126		*Myoviridae*	*S. aureus* 1S (MRSA) *S. aureus* 8S (MRSA) *S. aureus* 6M (MRSA) *S. aureus* 12M (MRSA)	MOI of 10 significantly reduced biofilm in all strains	Gharieb et al., 2020
	CF-301		*S. aureus* Newman *S. aureus* MW2 *S. aureus* CFS-1246 *S. aureus* VRS3a	MBEC-≤0.25 μg/ml	Schuch et al., 2017
UPKM_1		*Siphoviridae*	Active against 19 of 25 MRSA isolates		Dakheel et al., 2019
UPKM_2		*Podoviridae*	Active against 22 of 25 MRSA isolates		Dakheel et al., 2019
GH15	LysGH15	*Myoviridae*	*S. aureus* YB57 (MRSA)	All mice were rescued by 50 μg dose (*n* = 5)	Gu et al., 2011
SAP-2		*Podoviridae*	Active against 5 out 10 *S. aureus* strains tested		Son et al., 2010
	SAL-2		Active against 100% (10) of *S. aureus* strains tested against. Including MRSA	MIC – 1 μg/mL for MRSA isolates	Son et al., 2010
131			Active against 69% of 120 MRSA isolates tested		Pantucek et al., 2013
U16			Active against 65% of 120 MRSA isolates tested		Pantucek et al., 2013
SK311			Active against 52% of 120 MRSA isolates tested		Pantucek et al., 2013
Stau2		*Myoviridae*	*S. aureus* S23 *S. aureus* CS38 (MRSA) *S. aureus* KS7 (MRSA) *S. aureus* TS21 (MRSA)	Phage at an MOI of 10 administered immediately rescued 100% mice (*n* = 5) Reduced OD_600_ to nearly 0 after 3 h Reduced OD_600_ to nearly 0 after 3.5 h Reduced OD_600_ to nearly 0 after 3 h	Hsieh et al., 2011
	Lys109		*S. aureus* CCARM 3090 *S. aureus* RN4220	3 fold reduction in biofilm formation compared to SA012 (parental lysin)	Son et al., 2021
DMSA-2		*Siphoviridae*	*S. aureus* DSMA-2 (MRSA)	100% of mice in treatment group survived compared to 34% in the untreated group	Shetru et al., 2021

### Clinical Uses of Phages Against MRSA

The use of phage therapy in humans has mostly occurred in Eastern Europe and the former Soviet Union. In these cases, antibiotics may still be used as a first defense; however, upon failure to eradicate an infection, follow-up phage treatment has shown impressive results. Most studies reporting on the efficacy of phage treatment for bacterial infections report an ∼ 85% success rate (O’Flaherty et al., 2009). Although some studies have investigated the response of the immune system to phage treatment, results have been conflicting. For example, studies have reported that circulating phages in the blood elicit the innate immune system, while other studies have observed the ability of phages to go undetected by the immune system (Capparelli et al., 2007). Despite these conflicting results, clinical trials are underway with phages. Nine participants took part in a Phase I clinical trial, to examine the effect of the phage cocktail AB-SA01 on patients with recalcitrant chronic rhinosinusitis (Ooi et al., 2019). The results indicated the phage cocktail was well tolerated by patients with eradication of infection in two of the nine participants (Ooi et al., 2019).

Studies have also been published which describe the use of phage therapy as a compassionate treatment. The focus of compassionate treatment is not to provide results to evaluate the efficacy of the treatment but rather to benefit the patient when no other options are available. These case studies involving phage administration have indeed provided insight into the effectiveness of phage therapy in humans. Interestingly, in such cases of compassionate treatment, the most frequently reported bacterial infection to target is that of *S. aureus* (McCallin et al., 2019). It is important to note that compassionate treatment is generally only approved once all other treatments have been exhausted. One such example is a case study of six diabetic patients who underwent compassionate phage treatment for poorly perfused toe ulcers infected with *S. aureus* (Fish et al., 2016). The infection was non-responsive to antibiotic treatment and so therapy with staphylococcal phage Sb-1 took place. In all cases, phage treatment was sufficient to eradicate the *S. aureus* cells. Another case study gives an account of two individuals who obtained radiation burns and subsequently developed *S. aureus* infections in their wounds (Jikia et al., 2005). Following antibiotic therapy, the infection persisted in both cases. PhageBioDerm is a preparation comprising a cocktail of lytic phages with various specific hosts, in a biodegradable polymer mixture. The phage preparation inhibited the growth of *S. aureus* and eradicated the infection. The administration of PhageBioDerm in this case study demonstrates the non-invasive manner in which phages can be used to treat infection, in this case, applied as a film to the surface of the skin ulcer.

Depending on the type of infection with which a patient is suffering, varying methods of phage treatment may be selected. An infection site with a mixed community of pathogenic bacteria would respond better if treated with a phage cocktail. When choosing the most effective strategy for phage therapy, phage cocktails are widely considered the superior approach, allowing for higher and more prolonged bactericidal activity. This method of treatment consists of using a mixture of various phages with the same or different target hosts, depending on the type of infection. This creates a greater obstacle for the bacteria in terms of developing resistance (Tanji et al., 2004; Chan et al., 2013). Phage cocktails can also be used in combination with antibiotics. However, in such cases, the order of administration between the phage and antibiotics has rendered differential effects on the outcome of the treatment. Kumaran et al. (2018) carried out a study *in vitro* investigating the synergistic effect of lytic phage SATA-8505 with various antibiotics to inhibit biofilm-forming *S. aureus* cells. This study not only looked at the dual antimicrobial action of phages used in combination with antibiotics but also the order in which each therapeutic was administered. This study concluded that a treatment plan of phage therapy followed by antibiotic treatment proved the most effective at eradicating an *S. aureus* biofilm. Therefore, these results should be considered when designing a treatment plan involving both phage and antibiotic therapy (Abedon et al., 2011; Kumaran et al., 2018).

## Phage Lysins

### Background

Phages encode peptidoglycan hydrolases - highly evolved enzymes known as lysins that mediate the release of phage particles from bacterial host cells (Fischetti, 2010) and thus act from the inside out. Phages also encode virion-associated peptidoglycan hydrolases (VAPGH) - enzymes that mediate the injection of phage DNA into the bacterial cell by creating a small hole in the cell (Keary et al., 2014). Most endolysins have at least two functional domains, the N-terminal domain (location of enzymatic activity) and the cell wall binding domain (ensures enzymes act on their substrates) (Loessner, 2005). Phage endolysins induce bacterial lysis in Gram positive bacteria leading to cell death (Loessner, 2005; Fenton et al., 2010b). There are many advantages to using phage endolysins over antibiotics. Phage endolysins are specific for their target; therefore, collateral damage to commensal organisms will not occur. With regards to resistance, there are no known MRSA strains to date with resistance to phage lysins given that resistant strains would need to fundamentally change the structure of their cell wall. This highlights a huge benefit to using phage lysins to combat bacterial infections (Loeffler et al., 2001; Loessner, 2005). Additionally, phage lysins are thought to have the ability to counter bacterial resistance by targeting certain molecules that are key to maintaining cell viability (Garcia et al., 1983; Fischetti, 2010). The ability of phage lysins to infect and lyse MRSA strains have also been observed. Some examples of phage lysins eliciting such effects include phage endolysins MV-L (Rashel et al., 2007), Lys-SA012 (Fujiki et al., 2018), GH15 (Gu et al., 2011), PlySs2 (Gharieb et al., 2020), and LysK (O’Flaherty et al., 2005).

The development of lysins in recent years has come through three phases. 1^*st*^ generation lysins are natural lysins, which exhibit antimicrobial activity in their own right. An example of this is the phage lysin CF-301, produced by a *Streptococcus suis* prophage, which has demonstrated impressive anti-MRSA activity *in vitro* and *in vivo* (outlined below). CF-301 is also the first phage lysin to reach a phase III clinical trial (NCT04160468) (outlined below). 1^*st*^ generation lysins were improved upon, with the use of protein engineering, to produce 2^*nd*^ generation lysins (De Maesschalck et al., 2020), the goal of which was to enhance biochemical components and antimicrobial activity. 3^rd^ generation lysins are developed with the aim of optimization for clinical treatment in humans. This involves using protein and biochemical engineering, as well as innovative formulations to improve aspects including half-life, bioavailability, immune response and antimicrobial activity (De Maesschalck et al., 2020).

It is essential that lysins do not invoke a significant immune response. Strategies used in lysin engineering to escape the host immune response include glycosylation, PEGylation and T-cell epitope depletion (De Maesschalck et al., 2020). One example of a lysin designed to evade immune recognition is PEGylated lysostaphin (Walsh et al., 2003). Lysostaphin is a bacterially produced lytic enzyme. While this technique reduced the activity of the enzyme *in vitro*, the half-life of the modified lysostaphin *in vivo* was increased to 24 h compared with a half-life of less than 1 h for the unconjugated lysostaphin. As well as that, binding affinities for antibodies to the PEGylated lysostaphin were reduced by 10-fold compared with the unconjugated version. Reducing an immune response to lysins can also be achieved by formulations of lysins incorporated with anti-inflammatory compounds (Cheng et al., 2018). Due to the effect of renal filtration on the half-life of lysins, strategies that directly target this filtration process have been developed to improve lysin half-life. One strategy involves increasing hydrodynamic volume, by fusion of the chosen lysin and a water-binding polymer (Turecek et al., 2016). The lysin can also be dimerized in an attempt to reduce filtration (Grishin et al., 2019). While both these approaches did succeed in extending the half-life of the lysin, in both cases antimicrobial activity was negatively affected.

### Limitations to Phage Lysins

Lysins typically have a short half-life, this is due to glomerular filtration which is a system that removes small proteins (<40-50 kDa) from the body (De Maesschalck et al., 2020). This results in the majority of the lysin being excreted and subsequently unable to act on any infection present. Lysins are foreign proteins entering the host and so a pro-inflammatory response will be induced, involving production of anti-lysin antibodies (Schmelcher and Loessner, 2021). With the ability of *S. aureus* to persist inside eukaryotic cells, the inability of phage and most phage lysins to reach intracellular compartments of these eukaryotic cells makes complete eradication of the infections difficult (Schmelcher and Loessner, 2021). The majority of shortcomings of lysin treatment outlined above have been overcome or developed upon by the manufacturing of 2nd and 3rd generation lysins (discussed above).

### *In vitro* and *in vivo* Experiments of Phage Lysins Against MRSA

Nakamura et al. (2020) investigated the action of both phage phiSA012 and its lysin Lys-phiSA012 against multidrug-resistant staphylococcal isolates of canine origin. While the ability of the phage to lyse the staphylococcal isolates was not significant, the lytic activity of the lysin was far more impressive. Lys-phiSA012 lysed all multi drug resistant staphylococcal isolates except one, compared to the 4 multi drug resistant strains PhiSA012 was active against. Lys109 is a chimeric lysin with anti-staphylococcal activity. Crystal violet staining was used to examine the biofilm-inhibitory activity of the lysin, which reduced *S. aureus* CCARM 3090 and *S. aureus* RN4220 biofilms by 3-fold compared to its parental lysin SA012. At a concentration of 1000 nM Lys109 reduced the OD570 of the *S. aureus* CCARM 3090 and *S. aureus* RN4220 biofilms from 1 (control) to <0.2 in both cases (Son et al., 2021). CF-301 has also demonstrated impressive anti-biofilm activity *in vitro*. One study exhibited the ability of CF-301 to inhibit the growth of an *S. aureus* BAA-42 (MRSA) biofilm. This ability was further exhibited by the eradication of biofilms grown in synovial fluid and human blood, as well as biofilms grown under specific conditions favoring the growth of persistent *S. aureus* cells (Schuch et al., 2017). Another *in vitro* study used phage lysin to enhance a spider silk-based coating developed for orthopaedic and dental implants. The hypothesis is that this coating enhanced with Dispersin B and CF-301 or SAL1 will be able to reduce adhesion of bacteria causing common biomaterial-associated infection of orthopaedic and dental implants. The enhanced spider silk-based coating dramatically reduced attachment of bacteria and subsequent formation of biofilms (Nilebäck et al., 2019).

As previously discussed, *S. aureus* infection can take place at various locations throughout the body. This in itself leads to a variety of challenges in terms of the best route of delivery. Various *in vivo* studies have investigated the effects of phage endolysins on infections in a variety of locations throughout the body. The phage lysin CF-301 has shown impressive results for treating endocarditis and bloodstream infections *in vivo* (Schmelcher and Loessner, 2021). This lysin has also worked synergistically with a variety of antibiotics (Watson et al., 2020). Indiani et al. (2019) carried out a study utilizing a combination of CF-301 and daptomycin to treat endocarditis in rabbits. Treatment reduced the bacterial load of MRSA surrounding the heart by 6 logs. CF-301 has also been used to treat osteomyelitis, an infection of the bone commonly caused by *S. aureus* (Ellington et al., 2006). One week following infection, CF-301 and a combination of CF-301 and daptomycin were administered to rats intravenously. CF-301 did not remarkably reduce the bacterial load (0.48 logs), however, the combination treatment achieved slightly better results with a 1.56 log reduction in bacterial load (Karau et al., 2019). SAL200 is an endolysin produced by staphylococcal phage. The ability of SAL200 to treat MRSA-associated pneumonia in a mouse model has been investigated. The endolysin rescued 95-100% of mice following infection, whereas 40-93% of the control group died following 60 h of infection (Bae et al., 2019). Aquaphor gel was used to incorporate the lysin LysGH15 and apigenin, forming a LysGH15-api-Aquaphor ointment (Cheng et al., 2018). Apigenin is a plant-derived flavonoid with anti-inflammatory properties, which has been shown to reduce negative effects, such as hemolysis, caused by *S. aureus* infection in rabbits (De Maesschalck et al., 2020). This treatment improved healing of skin wounds in a murine model, while also decreasing levels of inflammatory cytokines IL-1β, TNF-α and IFN-γ (Cheng et al., 2018).

As described above, the ability of lysins to act on pathogenic bacteria present within eukaryotic cells is important for the inhibition of *S. aureus*. Becker et al. (2016) developed a 3^rd^ generation lysin engineered to inhibit intracellular *S. aureus*. Two parental proteins (LysK and lysostaphin) were fused to generate a recombinant protein with 3 antimicrobial activities. This fusion protein significantly reduced nasal colonization of S. *aureus* in a rat model (98% reduction), out-performing both parental proteins. The fusion protein was enhanced by addition of a protein transduction domain, which increased both inhibition of *S. aureus* biofilm as well as intracellular *S. aureus in vivo* (Becker et al., 2016). Röhrig et al. (2020) also used cell-penetrating peptide (CPP) activity to enhance selected lysins and deliver their antimicrobial action to intracellular compartments. This was achieved by the addition of a trans-activating transcription (tat) factor derived-CPP, which led to an intracellular MRSA reduction of up to 4.5 logs in various cells lines (A549, 3T3-L1, and MG-63) and more than 2.2 log reductions in a murine model with cutaneous abscesses (Röhrig et al., 2020). The phage lysin JDlys, produced by staphylococcal phage JD007, exhibited impressive anti-MRSA activity and so a study was performed to enhance the ability of JDlys to inhibit intracellular MRSA. Similar to the study just mentioned, tat factor-derived-CPPs were used for the enhancement of the lysin. This newly formed complex significantly reduced the presence of MRSA USA300 cells in HaCaT keratinocytes. This result was also achieved in a cutaneous abscess-induced mouse model, in which the CPP-JDlys protein reduced both bacterial load of the wound as well as the inflammatory response (Wang et al., 2018).

### Clinical Uses of Phage Lysins Against MRSA

SAL200 has shown promising results for treatment of blood stream infections, having already undergone studies to examine pharmacokinetics and safety in humans and pre-clinical characterization (NCT01855048) (Jun et al., 2017). SAL200 is currently in a Phase II clinical trial for treatment of blood stream infections (NCT03089697). Phage lysin CF-301 was used to treat endocarditis and blood stream infections in a Phase II clinical trial (NCT03163446). The study focused on cases of these diseases caused by *S. aureus*, with a subgroup of MRSA infection. Treatment solely with antibiotics was compared with a combination treatment of CF-301 and antibiotics. Combination treatment of individuals with MRSA infection resulted in a 74.1% response rate, compared to a response rate of 31.3% from antibiotic treatment alone (Fowler et al., 2020). CF-301 is currently undergoing a phase III clinical trial, in which CF-301 treatment of blood stream infections and endocarditis is being compared with antibiotic treatment in terms of efficacy and safety (NCT04160468). A biotechnology company, Micreos, has paved the way for the development of endolysin-centered products for human health by launching the brand Gladskin in 2019 (Herpers et al., 2016). One of their products is a recombinant endolysin, called Staphefekt SA.100, which when topically applied to the skin has been shown to dramatically reduce *S. aureus* infection and associated inflammation in patients (Totte et al., 2017). Staphefekt is, to date, the only commercially available phage preparation and is recognized in Europe as a Class I medical device which is available over the counter as a cetomacrogol-based gel or cream. The ability of the lysin to remain stable while also exhibiting such effective antimicrobial activities demonstrates potential for further development as a therapeutic for human health. A clinical trial examining the effect of Staphefekt on atopic dermatitis was recently performed (NCT02840955). The results of the twelve-week treatment showed that Staphefekt was well tolerated by patients (de Wit et al., 2019). Endolysin XZ.700, which is active against *S. aureus*, is a product that is also being developed by Micreos which has undergone pre-clinical trials (Herpers et al., 2016) and has successfully moved onto phase I/IIa clinical trial (NL8876).

## Conclusion

*Staphylococcus aureus* infections continue to be a challenge in hospital settings, particularly MRSA. This environment is primed for rapid infection spread due to the compromised immune response of patients and a large overlap between personnel and patient contacts. This is compounded by the phenotypic behavior of *S. aureus* and their propensity for forming biofilms on surgical equipment and colonizing open wounds in the skin. The rate at which bacterial strains and particularly staphylococci are developing immunity or resistance to antimicrobials is a major concern. New antibiotics are not being developed fast enough to counteract the antibiotic resistance crisis, with several existing antibiotics becoming ineffective in clinical practice (Field et al., 2016). Thus, development of antibiotic alternatives such as bacteriocins, phages and their endolysins are urgently needed to curb this increase in antibiotic resistance spread. The non-invasive manner in which these therapeutics can be applied, topically in a gel or cream form, adds to the attraction of using them to treat bacterial infections. When compared to antibiotics, bacteriocins and phages generally have narrow inhibition spectra, thus are target-specific, representing ideal therapeutics in an era when the importance of the microbiome to human health is being realized. Furthermore, due to their gene-encoded nature, bacteriocins can be bioengineered to produce variants with enhanced abilities. To date, bacteriocins such as nisin are being used for food safety and preservation purposes. However, it is envisaged that future bacteriocin research will focus on their development to treat infections in humans. This could focus on the synergistic effects of bacteriocins with antibiotic therapy, as well as innovative ways of administering bacteriocins to humans. Phage therapy is already established in certain parts of the globe but to gain acceptance in western medicine it will require more randomized controlled clinical trials and perhaps a new regulatory framework that can take full advantage of the potential that phage therapy has to offer. Phage endolysins are phage-derived enzymes and like their progenitor are target-specific. While bacterial cells can develop resistance to endolysins, resistance against currently developed endolysins for MRSA has not been documented, suggesting it is a challenging feat for the bacterial cell. Although there are limitations to both bacteriocin and phage therapy, these obstacles are being challenged by nanotechnology incorporated bacteriocins and 2^nd^ and 3^rd^ generation lysins. Going forward, phage- and bacteriocin-based therapies possess potential for the development of novel therapeutics which can replace and even enhance some existing antimicrobial therapies which are currently failing in the light of the antibiotic resistance crisis.

## Author Contributions

LW conceived and wrote the first draft of the manuscript. CJ, CH, and PR reviewed and contributed to the final manuscript. All authors contributed to the article and approved the submitted version.

## Conflict of Interest

The authors declare that the research was conducted in the absence of any commercial or financial relationships that could be construed as a potential conflict of interest.
